# Alterations in quality characteristics and bioactive compounds of blackberry fruits subjected to postharvest salicylic acid treatment during cold storage

**DOI:** 10.1002/fsn3.4490

**Published:** 2024-09-24

**Authors:** Mustafa Sakaldaş, Fatih Şen, Muttalip Gundogdu, Erdal Aglar

**Affiliations:** ^1^ Department of Food Processing, Vocational School of Lapseki Çanakkale Onsekiz Mart University Çanakkale Turkey; ^2^ Department of Horticulture, Faculty of Agriculture Ege University İzmir Turkey; ^3^ Department of Horticulture, Faculty of Agriculture Bolu Abant Izzet Baysal University Bolu Turkey; ^4^ Department of Horticulture, Faculty of Agriculture Van Yuzuncu Yıl University Van Turkey

**Keywords:** organic acids, phenolics, postharvest, salicylic, weight loss

## Abstract

Blackberry deteriorates rapidly after harvest due to its sensitive structure, limiting their storage time to about a week and resulting in significant economic losses. The study was conducted to determine the effects of salicylic acid applications on postharvest fruit quality in blackberries, the harvested fruit was immersed in salicylic acid solutions prepared at concentrations of 0.5, 1, and 1.5 mM for 15 min. Measurements and analyses such as weight loss, decay rate, soluble solids contents (SSC), pH, acidity, respiration rate, vitamin C, organic acids, and phenolic compounds were performed on fruits stored for 12 days with intervals of 4 days. Applying salicylic acid to fruits resulted in significantly less weight loss and decay rate. Salicylic acid application was effective in increasing SSC rate and decreasing titratable acidity with increasing storage time, and lower SSC and higher titratable acidity were measured with this application. Salicylic acid maintained organic acids and vitamin C postharvest. The decreases in individual phenolic compound levels occurred with extended storage time. Salicylic acid application generally was effective in maintaining concentrations of phenolic compounds during storage, and it was found to be effective on fruit quality, with effectiveness varying depending on application dosage. The study identified 1.5 mM as the most effective dosage of salicylic acid, which could be utilized to maintain postharvest quality and extend cold storage in blackberries.

## Graphical abstract

A study was conducted to assess the effects of salicylic acid on postharvest blackberry quality. Measurements such as weight loss, decay rate, soluble solids contents, pH, titratable acidity, respiration rate, vitamin C, organic acids, and phenolic compounds were taken over 12 days at 4‐day intervals. Salicylic acid‐treated fruits showed significantly less weight loss and decay. It also helped maintain organic acids, vitamin C, and phenolic compounds.

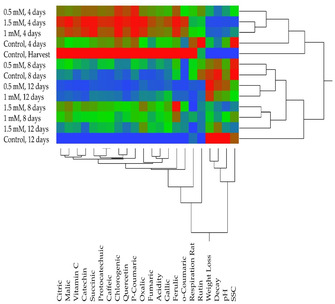

## 1 INTRODUCTION

Blackberries, rich in various antioxidants like benzoic acid, flavonoids, and anthocyanins, are excellent natural antioxidants and help prevent degenerative diseases (Carvalho & Betancour, 2015; Romero & Yepez, 2015). As a nonclimacteric fruit, blackberries quickly deteriorate due to high water content, delicate structure, and susceptibility to dehydration, injuries, and infections, resulting in a short postharvest life of 3–5 days (Ospina et al., 2019; Villegas & Albarracín, 2016). To extend the storage duration of blackberries, various practices have been implemented, such as the use of fungicides (Bersaneti et al., 2021), and thermal processes, which can adversely affect the fruit's antioxidant properties, nutritional content, and sensory characteristics (Villegas & Albarracín, 2016). In studies aimed at enhancing the preservation of products, it has been reported that the plant origin and variety of starch have a significant impact on the physical, mechanical, and thermal properties of starch‐based films (Pająk et al., 2019). Similarly, other studies with the same objective have investigated eco‐friendly and health‐conscious biodegradable applications containing substances such as *Aloe vera* (Ortega‐Toro et al., 2017), sodium alginate (Ruan et al., 2019), gellan gum (Du et al., 2019), gelatin (Jridi et al., 2019), casein (Chevalier et al., 2018), and hydroxypropyl methylcellulose (Villegas & Albarracín, 2016), and their contributions to their areas of use have been demonstrated.

Salicylic acid, a plant hormone, can modulate ethylene production or its effect, thereby slowing down the ripening process and potentially reducing deterioration and weight loss during storage. Salicylic acid, a simple phenolic compound with hormone‐like properties, regulates many metabolic and physiological processes related to plant growth, development, and stress response (Hayat et al., 2010). It is suggested that salicylic acid application during storage may enhance relevant gene expression and antioxidant capacities in fruits, triggering plant resistance against disease development and chilling damage, while maintaining quality characteristics such as sensory properties and nutritional content (Haider et al., 2020).

Studies show that salicylic acid effectively maintains postharvest fruit quality in species like blackberry, sweet cherry, banana, kiwi, apricot, grape, lemon, mandarin, and pear (Erbas et al., 2024; Haider et al., 2020; Sabir et al., 2019; Serna‐Escolano et al., 2021; Shi et al., 2019; Sinha et al., 2021; Yao & Tian, 2005; Zhang et al., 2003). However, there are few studies (Erbas et al., 2024; Sabir et al., 2019) regarding salicylic acid application in blackberries. Due to their sensitive structure, blackberries deteriorate rapidly after harvest, with a storage time of only 1 week. This poses a significant problem in terms of marketing and results in significant economic losses. In this context, the effects of postharvest salicylic acid applications on the quality characteristics and biochemical contents of blackberry fruits during storage were revealed in this study.

## 2 MATERIALS AND METHODS

The blackberry orchard row spacing is designed as 1.5 × 3 m. Irrigation is done using a drip irrigation system. The blackberry orchard is located in Düzce province (Türkiye) and has been established with the 6‐year‐old Jumbo cultivar. Handpicked blackberries (SSC 9%–10%) were placed in perforated plastic containers (four holes per unit area and 192 mm^2^ total area of holes) and transported the same day (August 25, 2023) in a refrigerated vehicle (4 ± 0.5°C and 90 ± 5% RH) to the Agriculture Faculty of Bolu Abant Izzet Baysal University. The remaining fruits were divided into four groups (control, 0.5, 1, and 1.5 mM), with each group comprising 60 fruits for each application. One group was the control while fruits from the other three groups were immersed in salicylic acid solutions prepared at different concentrations for 15 min. Fruits were immersed in salicylic acid solutions with 0.2% Tween‐20 for 15 min; the control group was soaked in distilled water (15 min). After salicylic acid treatment (control, 0.5, 1, and 1.5 mM), fruits in plastic containers (375‐g capacity, width: 110 mm, length: 120 mm, height: 253 mm) were stored at 0 ± 0.5°C and 90 ± 5% RH. Measurements and analyses were conducted every 4 days over 12 days.

### 2.1 Weight loss

After salicylic acid application, the initial weight (*W*_i_) of the fruits (500 g) was measured with a digital scale (precision 0.01 g). During 12 days of storage, weights (*W*_f_) were measured on the 4th, 8th, and 12th days. Weight loss was calculated using Equation (1).

(1) $$\mathrm{WL}=\frac{W_{i}-W_{f}}{W_{i}}\times 100$$

### 2.2 Decay rate

After the salicylic acid application, the number of the fruits in each replicate (15 fruits) and the total number of fruits for each application (60 fruits) were recorded (TF). In fruits stored for 12 days, on the 4th, 8th, and 12th days of the cold storage, the rotten fruits in each repetition and in each application were detected by including the rotten fruits in those with mycelial development on the peel. The decay rate (DR) in the treatments was recorded as a percentage using the following Equation (2).

(2) $$\mathrm{DR}=\frac{\mathrm{TF}-\mathrm{DF}}{\mathrm{TF}}\times 100$$

### 2.3 Total soluble solid content (SSC), pH, and titratable acidity (TA)

During measurement periods, fruits were washed with distilled water, homogenized, and passed through cheesecloth to obtain juice for SSC and TA measurements. SSC was measured with a digital refractometer (Atago PAL‐1) and recorded as a percentage. pH was measured with a pH meter. For TA, 10 mL of juice was diluted with distilled water and titrated with 0.1 N NaOH to a pH of 8.2. TA was recorded as citric acid (%).

### 2.4 Respiration rate

Respiration rate measurements were conducted using airtight chambers with a volume of 2 L. Each chamber, equipped with a rubber septum, enclosed 80 fruits and was maintained at 21 ± 1°C and 90% RH for an hour. Subsequently, the chambers were linked to a gas analyzer (Vernier, Oregon, USA) to quantify the amount of CO_2_ emitted by the fruits, indicating their respiration rate. The findings were reported as milligrams of CO_2_ produced per kilogram per hour.

### 2.5 Organic acids

Organic acids were extracted from fresh samples using a modified method by Bevilacqua and Califano (1989). Fifty grams of sample was treated with 10 mL of 0.009 N H_2_SO_4_, homogenized, and centrifuged at 14,000 rpm for 15 min. The supernatant was filtered and passed through a SEP‐PAK C18 cartridge before HPLC analysis (Agilent HPLC 1100 series). Organic acids were identified using an Aminex HPX‐87H column at 210 nm with 0.009 N H_2_SO_4_ as the mobile phase.

### 2.6 Phenolic compounds

For phenolic compound analysis, 250 g of fruit was homogenized and mixed with distilled water (1:1 ratio). The mixture was centrifuged at 15,000 rpm for 15 min. Supernatants were filtered through coarse filter paper and then through a 0.45‐μm membrane filter (Millipore Millex‐HV Hydrophilic PVDF, USA) before injection into an Agilent HPLC system. Chromatographic separation used a 250 × 4.6 mm, 4 μm ODS column (HiChrom, USA) with a mobile phase of solvent A (methanol:acetic acid:water—10:2:28) and solvent B (methanol:acetic acid:water—90:2:8). Detection wavelengths were 270 and 280 nm. The flow rate was 1 mL/min, and 20 μL was injected. Standards were from Sigma‐Aldrich (Germany) with ≥99% purity (Rodríguez‐Delgado et al., 2001).

### 2.7 Statistical analysis

In this study, data analysis utilized SAS Version 9.1 (SAS Institute Inc., Cary, NC, USA) for two‐way ANOVA and Tukey's post hoc test for significant results. Pearson's pairwise correlations were computed using the “corrplot” package in R (Wei et al., 2017). Principal component analysis (PCA) was conducted with the “ggplot2” package in R to explore interactions between storage periods, spermidine doses, and various traits. Additionally, data visualization included heatmap analysis using the “bioconductor” package in R (Gentleman et al., 2004).

## 3 RESULTS AND DISCUSSION

### 3.1 Quality properties

**FIGURE 1:**
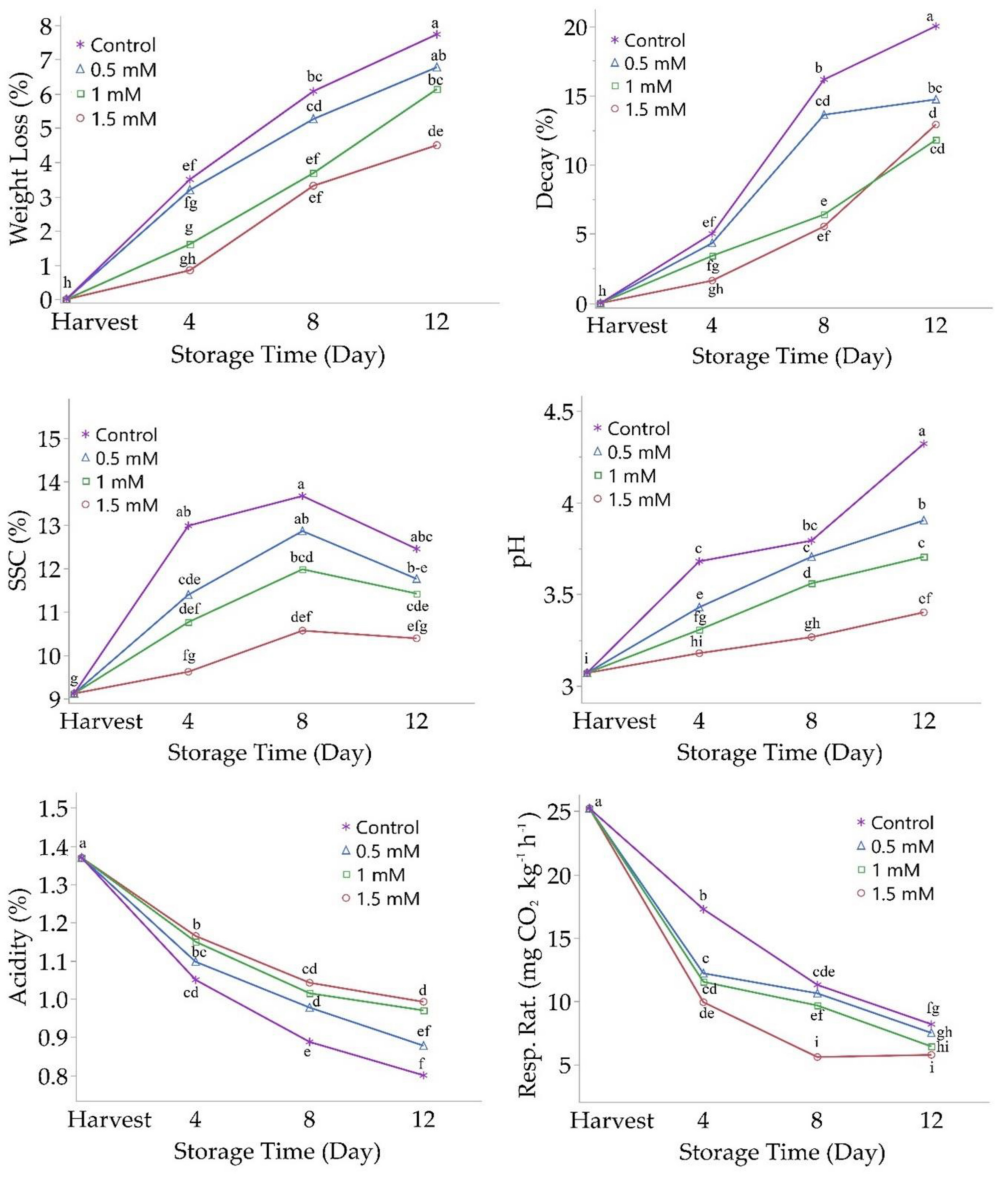
The effect of salicylic acid on the change in quality characteristics of blackberry fruits during storage. Distinct letterings above the storage periods indicate statistically significant differences at *p* ≤ .05.

Weight loss in the fruits significantly increased over storage time, from 2.29% on the 4th day to 6.28% by the end of storage (Figure 1; Table S1). The results of the study clearly demonstrate the effect of salicylic acid application on weight loss during storage. Applying salicylic acid to fruits resulted in significantly less weight loss. The weight loss in fruits treated with salicylic acid was significantly lower compared to the control group. Furthermore, the concentration of salicylic acid application was found to have a significant effect on reducing weight loss, with the effectiveness increasing with higher concentrations. A weight loss of 5.07% was recorded with 0.5 mM salicylic acid application, and weight loss of 2.89% was observed with 1.5 mM salicylic acid application. When the interaction between storage time and salicylic acid application was evaluated, salicylic acid application was effective in reducing weight loss throughout all measurement periods as storage time increased, and its effectiveness varied depending on the applied concentration. The lowest weight loss values were obtained with the application of 1.5 mM salicylic acid. Similar weight loss was observed in the control group and fruits treated with 0.5 mM salicylic acid throughout all measurement periods while there was no difference in weight loss between fruits treated with 1.5 mM salicylic acid and the control group fruits on the 4th day of storage. These findings indicate that salicylic acid application reduces water loss in blackberries, thereby aiding in reducing weight loss during storage. Weight loss is a significant factor affecting the external appearance, texture, and perceived freshness of fruits during storage. Reducing this loss through salicylic acid application allows for the extension of the storage time of blackberries and provides consumers with fresher and higher quality fruits. In conclusion, salicylic acid plays a significant role in influencing the effects of storage time and their interaction, with storage time being the more pronounced factor.

The decay rate in fruits increased proportionally with storage time. The decay rate, which was 3.60% on the 4th day of storage, was recorded as 14.85% by the end of storage. However, it was found that salicylic acid application was effective in reducing the decay rate, with significantly lower decay occurring in fruits treated with salicylic acid (Figure 1; Table S1). The changes in the decay rate occurred depending on the concentration of salicylic acid application, but the effectiveness increased with higher concentrations. A decay rate of 10.88% was observed in fruits treated with 0.5 mM salicylic acid, decay rate of 6.69% was recorded with the application of 1.5 mM salicylic acid. When the interaction between storage time and salicylic acid application was evaluated, it was observed that salicylic acid application was effective in reducing decay throughout all measurement periods as storage time increased, and its effectiveness varied depending on the applied concentration. On the 4th and 8th days of storage, fruits treated with 1.5 mM salicylic acid exhibited less decay compared to other treatments, while by the end of storage (12th day), the 1 mM treatment was found to be more effective in reducing the decay rate (Table S1). Consequently, it can be said that salicylic acid plays a significant role in influencing the effects of storage time and their interaction, with storage time being the more pronounced factor. Weight loss in fruits postharvest is influenced by metabolic changes like respiration and transpiration, leading to significant economic losses (Madhav et al., 2021). As demonstrated in this study, weight loss and decay rate increase with extended storage time (Rastegar et al., 2020). Salicylic acid, a natural and safe phenolic compound, can mitigate weight loss by reducing metabolic activity and transpiration in fruits (Amiri et al., 2021). It is suggested that salicylic acid enhances plant resistance against diseases and chilling damage, boosting gene expression and antioxidant capacity in stored fruits, thereby preserving sensory qualities (flavor, appearance, texture) and nutritional compounds (García‐Pastor et al., 2020). Previous studies have shown that salicylic acid application reduces weight loss and decay in various fruits during storage, such as banana, Barhi date, lemon, papaya, litchi, grape, apricot, plum, cherry, orange, and pomegranate (Amiri et al., 2021; Atia et al., 2018; Batool et al., 2022; Davarynejad et al., 2015; García‐Pastor et al., 2020; Gimenez et al., 2016; Hanif et al., 2020; Hazarika & Marak, 2021; Kumar et al., 2013; Serna‐Escolano et al., 2021; Xu et al., 2019). However, the effectiveness of salicylic acid in maintaining fruit quality and reducing weight loss can vary based on factors like concentration, application method, variety, and storage conditions.

There was a significant effect of storage time on SSC (soluble solids content) in blackberries, and generally, an increase in SSC occurred as the storage time increased, with the highest SSC value recorded on the 8th day of storage (Figure 1; Table S1). However, salicylic acid application was effective in reducing the increase in SSC ratio with the extension of storage time, resulting in lower values with this application. The effect varied depending on the concentration of salicylic acid application, with SSC ratio decreasing as the application concentration increased. Salicylic acid, a plant hormone, can slow down ripening and reduce spoilage and weight loss during storage by regulating the production or effects of ethylene. In addition, it reduces water loss in fruits and preserves biochemical changes (Hayat et al., 2010). This result can be explained by the salicylic acid's effect of limiting water loss and biochemical changes in blackberry fruits during storage, as well as its antifungal properties. When the interaction between salicylic acid and storage time was evaluated in terms of SSC (soluble solid content), there was no difference between the SSC values measured in fruits treated with 1.5 mM salicylic acid during storage and the SSC value recorded at harvest. Consequently, it can be concluded that the application of 1.5 mM salicylic acid is the most effective method in slowing down the deterioration of fruits and therefore in preserving fruit quality. The highest values were obtained in the control group fruits on all measurement days, and generally, there was no difference between 0.5 and 1 mM salicylic acid treatments. In conclusion, salicylic acid plays an important role in affecting the impacts of storage duration and its interactions, and salicylic acid is a more prominent factor.

There was an increase in pH values and a decrease in TA, reflecting the progression of ripening as storage time increased. However, salicylic acid application affected the changes in pH and TA during storage (Figure 1). Despite the higher pH and lower A measured during storage in fruits treated with salicylic acid compared to the values measured at harvest, TA ratios were higher than those in the control group, and A values increased with the concentration of salicylic acid. When the interaction between salicylic acid and storage time was evaluated, the lowest pH and highest A values during storage were obtained with the application of 1.5 mM salicylic acid, and significant differences were observed between applications and storage days. Salicylic acid and storage time play important roles in influencing the results in terms of pH, with salicylic acid application having a more pronounced effect, and the combination of the two further enhancing the effect. In terms of TA values, salicylic acid and storage time also have an important impact on the results, but the effect of storage time is greater.

The SS/A ratio, an indicator of fruit taste and ripeness (Hazarika & Marak, 2021), increases with ripening due to rising SSC and declining TA. Preventing this increase is crucial for extending storage time. Salicylic acid can suppress this rise by affecting respiration and metabolic activity (Champa et al., 2014). Kumar et al. (2013) observed a decrease in SSC/TA ratio in litchi fruits treated with salicylic acid. SSC decrease during storage is primarily due to respiratory metabolism (Hazarika & Marak, 2021). Starch and other insoluble carbohydrates hydrolyze into soluble sugars during fruit ripening, increasing respiration rate and SSC (Haider et al., 2021). Salicylic acid applications have maintained SSC levels in fruits like banana, Barhi date, papaya, litchi, grape, and apricot during storage (Atia et al., 2018; Batool et al., 2022; Hanif et al., 2020; Hazarika & Marak, 2021; Kumar et al., 2013; Xu et al., 2019). Kumar et al. (2021) noted that salicylic acid slowed SSC increase in tomatoes, attributing it to reduced fruit metabolic activity and hydrolysis of complex carbohydrates to SSC. TA, positively correlated with organic acid content, decreases with prolonged storage due to pyruvate decarboxylation during ripening (Hazarika & Marak, 2021). Salicylic acid applications have slowed TA decrease during storage in oranges, sweet cherry, and apricot (Amiri et al., 2021; Fan et al., 2021; Gimenez et al., 2016). In grapes, however, lower TA compared to the control suggests a significant interaction between salicylic acid and storage duration on TA, with storage time having a greater impact (Hazarika & Marak, 2021).

The respiration rate of fruits significantly decreased with increased storage time. Initially, at 25.22 mg CO_2_ kg^−1^ h^−1^ at harvest, it decreased to 6.97 mg CO_2_ kg^−1^ h^−1^ by the end of storage. Salicylic acid application notably reduced the respiration rate, with effectiveness increasing alongside higher application concentrations. For instance, respiration rates were 10.11 CO_2_ kg^−1^ h^−1^ with 0.50 mM salicylic acid and 7.09 mg CO_2_ kg^−1^ h^−1^ with 1.50 mM salicylic acid. Throughout storage periods, salicylic acid consistently reduced the respiration rate, with the most pronounced effect observed at 1.5 mM concentration. These findings underscore the effectiveness of salicylic acid in reducing fruit respiration during storage, influenced significantly by both salicylic acid concentration and storage time. Respiration, a fundamental physiological process providing energy for metabolism, is regulated by ethylene in fruits (Kumar et al., 2021). Salicylic acid suppresses ethylene‐related gene expression, such as 1‐aminocyclopropane‐1‐carboxylic acid oxidase and synthase, thereby delaying ethylene production, slowing the respiration rate, and delaying fruit aging in species like tomatoes, grapes, asparagus, and plums (Champa et al., 2014; Kumar et al., 2021; Luo et al., 2012; Wei et al., 2011).

### 3.2 Organic acids and vitamin C

In the study, blackberries exhibited the highest levels of citric acid, followed by malic, succinic, oxalic, and fumaric acids in terms of quantity. As storage time increased, there was a decrease in the amounts of all organic acids. Salicylic acid application effectively preserved organic acid levels during storage, with no significant differences generally observed among application concentrations. However, fruits treated with 0.5 mM salicylic acid showed higher amounts of fumaric and oxalic acids compared to the control, yet relatively lower than the other two application rates. Similarly, there were no significant differences between the control and 0.5 mM applications in terms of citric, malic, and succinic acid levels (Table 1). Evaluation of the interaction between storage time and salicylic acid application revealed that salicylic acid influenced the levels of all organic acids throughout the measurement periods as storage time increased, with varying effects depending on the applied concentration. The highest values were observed with the application of 1.5 mM salicylic acid. These findings indicate the role of salicylic acid application in preserving the quantity of organic acids in blackberries, with both salicylic acid and storage time playing a significant role in the changes in organic acid quantity, but the effect of storage time being more pronounced, and their combination further enhancing the effect. Salicylic acid, a hormone that plays important roles in plant growth, development, flowering, fruiting, and increasing resistance to disease and stress conditions, triggers plant defense mechanisms and activates different biochemical pathways when applied externally to plants (Hayat et al., 2010). By reducing the respiration rate in fruits, affecting enzyme activities involved in the synthesis of some organic acids, and making fruits more resistant to abiotic stress conditions such as cold and drought, as well as pathogens, salicylic acid can contribute to the preservation of organic acid content (García‐Pastor et al., 2020). Indeed, Sayyari et al. (2011) and Kazemi et al. (2011) reported the effectiveness of salicylic acid application in maintaining postharvest organic acids, although it should be noted that the effect of application may vary depending on fruit type, application timing, dose, and storage conditions.

**TABLE 1:** Impact of salicylic acid dosages on organic acid (mg g^−1^) and vitamin C (mg 100 g^−1^) contents of blackberry fruit throughout storage periods.

Salicylic acid application	Citric	Malic	Succinic	Oxalic	Fumaric	Vitamin C
Control	3.96 ± 0.14b	2.68 ± 0.08b	1.47 ± 0.11b	0.84 ± 0.06c	0.44 ± 0.03c	14.41 ± 0.49b
SA 0.5 mM	4.14 ± 0.13ab	2.81 ± 0.08ab	1.63 ± 0.10ab	1.02 ± 0.05b	0.58 ± 0.05b	15.42 ± 0.58ab
SA 1 mM	4.24 ± 0.13ab	2.92 ± 0.08a	1.79 ± 0.10a	1.11 ± 0.05ab	0.65 ± 0.05ab	15.95 ± 0.57a
SA 1.5 mM	4.39 ± 0.12a	3.01 ± 0.07a	1.90 ± 0.09a	1.24 ± 0.03a	0.74 ± 0.04a	16.65 ± 0.47a
Storage time						
Harvest	5.15 ± 0.03a	3.37 ± 0.02a	2.36 ± 0.07a	1.44 ± 0.03a	1.14 ± 0.03a	20.17 ± 0.21a
Day 4	4.60 ± 0.04b	3.08 ± 0.04b	2.06 ± 0.04b	1.22 ± 0.03b	0.76 ± 0.04b	17.47 ± 0.26b
Day 8	4.24 ± 0.05c	2.92 ± 0.04c	1.65 ± 0.05c	1.02 ± 0.05c	0.56 ± 0.03c	15.48 ± 0.26c
Day 12	3.71 ± 0.06d	2.58 ± 0.05d	1.39 ± 0.06d	0.90 ± 0.06c	0.48 ± 0.03c	13.88 ± 0.27d
Storage time × Salicylic acid interaction						
Harvest	5.15 ± 0.03	3.37 ± 0.02	2.36 ± 0.07	1.44 ± 0.03	1.14 ± 0.03	20.17 ± 0.21
Day 4						
Control	4.45 ± 0.03d	2.90 ± 0.04d	1.86 ± 0.05d	1.08 ± 0.02ef	0.56 ± 0.02ef	16.18 ± 0.19de
SA 0.5 mM	4.56 ± 0.02c	3.04 ± 0.03c	2.00 ± 0.03c	1.19 ± 0.03c	0.77 ± 0.03cd	17.39 ± 0.21c
SA 1 mM	4.62 ± 0.01c	3.15 ± 0.03b	2.16 ± 0.01b	1.29 ± 0.01b	0.83 ± 0.03bc	18.02 ± 0.05b
SA 1.5 mM	4.79 ± 0.03b	3.22 ± 0.03b	2.20 ± 0.05b	1.34 ± 0.04b	0.89 ± 0.03b	18.29 ± 0.16b
Day 8						
Control	3.98 ± 0.03g	2.74 ± 0.02e	1.44 ± 0.04hi	0.77 ± 0.04i	0.42 ± 0.02g	14.18 ± 0.14h
SA 0.5 mM	4.20 ± 0.04f	2.91 ± 0.02d	1.55 ± 0.02fg	1.01 ± 0.02fg	0.53 ± 0.02f	15.48 ± 0.10fg
SA 1 mM	4.32 ± 0.02e	2.99 ± 0.02cd	1.73 ± 0.03e	1.10 ± 0.03de	0.58 ± 0.01ef	15.75 ± 0.13ef
SA 1.5 mM	4.45 ± 0.03d	3.04 ± 0.03c	1.88 ± 0.03d	1.20 ± 0.04c	0.72 ± 0.01d	16.50 ± 0.27d
Day 12						
Control	3.45 ± 0.04j	2.41 ± 0.05g	1.11 ± 0.03j	0.66 ± 0.04j	0.34 ± 0.01h	12.86 ± 0.25j
SA 0.5 mM	3.67 ± 0.01i	2.49 ± 0.04g	1.34 ± 0.02i	0.85 ± 0.01hi	0.45 ± 0.01g	13.40 ± 0.15i
SA 1 mM	3.77 ± 0.01h	2.62 ± 0.03f	1.50 ± 0.03gh	0.93 ± 0.02gh	0.53 ± 0.02f	14.09 ± 0.09h
SA 1.5 mM	3.95 ± 0.04g	2.78 ± 0.02e	1.63 ± 0.04ef	1.17 ± 0.02cd	0.61 ± 0.01e	15.15 ± 0.23g
ANOVA						
*F* (Salicylic acid)	1.92^ns^	3.49*	3.75*	10.85***	8.14***	3.17*
*F* (Storage time)	87.65***	41.81***	40.24***	13.82***	31.71***	57.01***
*F* (Salicylic acid × Storage time)	270.54***	82.58***	91.47***	62***	88.29***	136.67***

*Note*: Different letters in the same column indicate statistical differences at *p* ≤ .05.

Abbreviation: ns, not significant.

* *p* ≤ .05.

*** *p* ≤ .001.

Storage time significantly decreased vitamin C content, with the lowest levels observed at the end of storage. Salicylic acid application effectively mitigated this decline, resulting in higher vitamin C levels compared to the control. Different concentrations of salicylic acid showed no significant differences in their effect. Overall, both salicylic acid and storage time influenced outcomes, but storage duration had a more pronounced effect. Fruits and vegetables are natural sources of vitamin C, a potent antioxidant crucial for human health. Postharvest, factors like high temperature and low humidity can reduce vitamin C content. Salicylic acid application helps maintain or increase vitamin C levels by enhancing antioxidant defense systems, boosting ascorbate peroxidase activity, and reducing ascorbic acid oxidation (Haider et al., 2020). El‐Shazly et al. (2013) demonstrated reduced ascorbic acid loss in pineapples with salicylic acid application, while Wei et al. (2011) found salicylic acid preserved phenolic compounds, flavonoids, and ascorbic acid in asparagus plants.

### 3.3 Phenolic compounds

The individual phenolic compound found in the highest quantity in blackberries was catechin, followed by rutin, gallic acid, caffeic acid, quercetin, chlorogenic acid, *p*‐coumaric acid, protocatechuic acid, ferulic acid, and *o*‐coumaric acid, respectively. With the extension of storage time, decreases in the quantities of these individual phenolic compounds occurred. Salicylic acid application was generally observed to be effective in maintaining the concentrations of phenolic compounds during storage (Tables 2 and 3). However, the differences in the effect were observed depending on the compound. For example, in fruits treated with salicylic acid, concentrations of compounds such as caffeic acid, catechin, chlorogenic acid, protocatechuic acid, and ferulic acid were higher during storage compared to the control, with no differences observed among salicylic acid application concentrations. However, the concentration of rutin was higher in the control group fruits, with no differences observed among other applications. Salicylic acid application was effective on gallic acid and *o*‐coumaric acid, and the effect varied depending on the application concentration, with the highest values recorded in fruits treated with 1.5 mM salicylic acid. However, no effect of salicylic acid application was observed on quercetin and *p*‐coumaric acid concentrations. Salicylic acid and storage time were found to play a significant role in influencing the outcome in terms of phenolic compounds, with the effect of storage time being more pronounced, and their combination further enhancing the effect. Phenolic compounds play a crucial role in reducing the accumulation of reactive oxygen species (ROS) such as hydroxyl radicals, superoxide, and hydrogen peroxide, which cause oxidative damage, cell death, and tissue damage during fruit ripening. This helps preserve fruit aroma and quality (Champa et al., 2014). Salicylic acid application supports the maintenance of phenolic compounds during storage, enhancing antioxidant capacity to reduce oxidative damage, prolong storage time, and delay fruit aging in fruits such as sweet cherry, pomegranate, lemon, and plum (Davarynejad et al., 2015; García‐Pastor et al., 2020; Serna‐Escolano et al., 2021; Valverde et al., 2015). Salicylic acid has also been shown to increase enzyme activities like phenylalanine ammonia‐lyase, cinnamate 4‐hydroxylase, and 4‐coumarate‐coenzyme A ligase, thereby boosting phenolic compound content in fruits such as oranges and grapes (Amiri et al., 2021; Blanch et al., 2020). Additionally, it prevents anthocyanin breakdown by polyphenol oxidase (PPO) enzymes in blood oranges (Habibi et al., 2020). Previous studies have consistently reported that salicylic acid maintains antioxidant activity, phenolics, and bioactive compound content in fruits such as bamboo, plum, pomegranate, sweet cherry, mandarin, grape, and oranges (Baswal et al., 2020; Davarynejad et al., 2015; Gomes et al., 2021; Habibi et al., 2020; Koyuncu et al., 2019; Luo et al., 2012; Valero et al., 2011).

**TABLE 2:** Impact of salicylic acid dosages on phenolic compounds (mg 100 g^−1^) of blackberry fruit throughout storage periods.

Salicylic acid application	Caffeic	Catechin	Chlorogenic	Ferulic	Gallic
Control	1.45 ± 0.15b	143.33 ± 4.60b	1.24 ± 0.06b	0.83 ± 0.03b	3.79 ± 0.09b
SA 0.5 mM	1.72 ± 0.13ab	150.67 ± 5.98ab	1.29 ± 0.06ab	0.86 ± 0.03ab	3.92 ± 0.07b
SA 1 mM	1.84 ± 0.11a	156.67 ± 6.26ab	1.35 ± 0.05ab	0.90 ± 0.03ab	4.12 ± 0.04a
SA 1.5 mM	1.98 ± 0.10a	162.87 ± 6.51a	1.40 ± 0.05a	0.93 ± 0.02a	4.20 ± 0.06a
Storage time					
Harvest	2.65 ± 0.07a	193.71 ± 2.61a	1.64 ± 0.02a	1.01 ± 0.01a	4.71 ± 0.11a
Day 4	2.17 ± 0.04b	173.25 ± 2.99b	1.53 ± 0.01b	0.95 ± 0.01b	4.23 ± 0.04b
Day 8	1.69 ± 0.08c	153.60 ± 2.40c	1.27 ± 0.02c	0.92 ± 0.01b	3.99 ± 0.05c
Day 12	1.38 ± 0.08d	133.30 ± 1.59d	1.17 ± 0.02d	0.77 ± 0.02c	3.81 ± 0.07d
Storage time × Salicylic acid interaction					
Harvest	2.65 ± 0.07a	193.71 ± 2.61a	1.64 ± 0.02a	1.01 ± 0.01a	4.71 ± 0.11a
Day 4					
Control	2.01 ± 0.05de	158.40 ± 1.39f	1.47 ± 0.01c	0.91 ± 0.01de	4.10 ± 0.03de
SA 0.5 mM	2.13 ± 0.05cd	171.80 ± 1.96d	1.51 ± 0.02c	0.94 ± 0.01c	4.16 ± 0.02cd
SA 1 mM	2.20 ± 0.03bc	178.80 ± 1.85c	1.56 ± 0.01b	0.96 ± 0.01bc	4.26 ± 0.04bc
SA 1.5 mM	2.31 ± 0.04b	184.00 ± 1.62b	1.59 ± 0.01b	0.97 ± 0.01b	4.38 ± 0.08b
Day 8					
Control	1.27 ± 0.05i	144.60 ± 1.04g	1.20 ± 0.02f	0.87 ± 0.01f	3.80 ± 0.06g
SA 0.5 mM	1.74 ± 0.07fg	149.40 ± 1.50g	1.24 ± 0.01ef	0.90 ± 0.01e	3.87 ± 0.04fg
SA 1 mM	1.83 ± 0.05fg	155.20 ± 2.31f	1.28 ± 0.01e	0.94 ± 0.01cd	4.10 ± 0.04cde
SA 1.5 mM	1.91 ± 0.05ef	165.20 ± 0.69e	1.36 ± 0.03d	0.97 ± 0.01b	4.18 ± 0.01cd
Day 12					
Control	1.05 ± 0.06j	127.00 ± 1.73i	1.06 ± 0.01h	0.71 ± 0.01j	3.47 ± 0.07h
SA 0.5 mM	1.27 ± 0.03i	130.80 ± 1.39i	1.13 ± 0.02g	0.74 ± 0.01i	3.73 ± 0.03g
SA 1 mM	1.48 ± 0.03h	136.00 ± 1.39h	1.21 ± 0.01f	0.79 ± 0.01h	3.99 ± 0.02ef
SA 1.5 mM	1.72 ± 0.12g	139.40 ± 1.96h	1.26 ± 0.02e	0.84 ± 0.01g	4.05 ± 0.05de
ANOVA					
*F* (Salicylic acid)	3.55*	2.01^ns^	1.57^ns^	2.19^ns^	7.74***
*F* (Storage time)	37.08***	70.93***	84.26***	52.24***	21.57***
*F* (Salicylic acid × Storage time)	60.99***	151.89***	139.1***	91.2***	32.85***

*Note*: Different letters in the same column indicate statistical differences at *p* ≤ .05.

Abbreviation: ns, not significant.

* *p* ≤ .05.

*** *p* ≤ .001.

**TABLE 3:** Continuation of Table 2 (mg 100 g^−1^).

Salicylic acid application	*o*‐Coumaric	*p*‐Coumaric	Protocatechuic	Quercetin	Rutin
Control	0.43 ± 0.01c	1.00 ± 0.05a	0.62 ± 0.06b	1.51 ± 0.06a	5.90 ± 0.29a
SA 0.5 mM	0.46 ± 0.02bc	1.05 ± 0.04a	0.73 ± 0.05ab	1.58 ± 0.07a	5.50 ± 0.12ab
SA 1 mM	0.54 ± 0.03ab	1.10 ± 0.04a	0.78 ± 0.04a	1.63 ± 0.06a	5.27 ± 0.06b
SA 1.5 mM	0.59 ± 0.04a	1.12 ± 0.04a	0.84 ± 0.04a	1.68 ± 0.06a	5.27 ± 0.04b
Storage time					
Harvest	0.89 ± 0.02a	1.27 ± 0.03a	1.13 ± 0.03a	2.08 ± 0.01a	5.34 ± 0.12bc
Day 4	0.61 ± 0.03b	1.21 ± 0.01a	0.92 ± 0.02b	1.85 ± 0.02b	5.88 ± 0.17a
Day 8	0.47 ± 0.02c	1.06 ± 0.01b	0.72 ± 0.03c	1.56 ± 0.03c	5.53 ± 0.11b
Day 12	0.44 ± 0.01c	0.92 ± 0.02c	0.59 ± 0.03d	1.40 ± 0.02d	5.05 ± 0.05c
Storage time × Salicylic acid interaction					
Harvest	0.89 ± 0.02a	1.27 ± 0.03a	1.13 ± 0.03a	2.08 ± 0.01a	5.34 ± 0.12def
Day 4					
Control	0.48 ± 0.01e	1.16 ± 0.01c	0.86 ± 0.02cd	1.76 ± 0.01d	6.81 ± 0.06a
SA 0.5 mM	0.54 ± 0.01d	1.20 ± 0.01bc	0.90 ± 0.02c	1.85 ± 0.02c	5.83 ± 0.05c
SA 1 mM	0.67 ± 0.01c	1.23 ± 0.01ab	0.93 ± 0.02bc	1.85 ± 0.01c	5.48 ± 0.03d
SA 1.5 mM	0.74 ± 0.02b	1.25 ± 0.01a	0.98 ± 0.02b	1.92 ± 0.01b	5.39 ± 0.03de
Day 8					
Control	0.42 ± 0.01fg	1.00 ± 0.00ef	0.54 ± 0.02h	1.44 ± 0.01i	6.05 ± 0.04b
SA 0.5 mM	0.43 ± 0.01fg	1.06 ± 0.02d	0.73 ± 0.03f	1.52 ± 0.01g	5.66 ± 0.03c
SA 1 mM	0.49 ± 0.02e	1.10 ± 0.01d	0.78 ± 0.02ef	1.61 ± 0.02f	5.18 ± 0.05fgh
SA 1.5 mM	0.55 ± 0.02d	1.10 ± 0.01d	0.82 ± 0.02de	1.65 ± 0.01e	5.24 ± 0.06efg
Day 12					
Control	0.40 ± 0.02g	0.84 ± 0.02h	0.44 ± 0.03i	1.33 ± 0.01	4.83 ± 0.09i
SA 0.5 mM	0.42 ± 0.03fg	0.90 ± 0.01g	0.54 ± 0.01h	1.37 ± 0.01j	5.03 ± 0.07h
SA 1 mM	0.46 ± 0.01ef	0.96 ± 0.01f	0.63 ± 0.01g	1.43 ± 0.01i	5.15 ± 0.03gh
SA 1.5 mM	0.49 ± 0.01e	1.01 ± 0.01e	0.72 ± 0.05f	1.48 ± 0.01h	5.18 ± 0.05fgh
ANOVA					
*F* (Salicylic acid)	6.33**	1.57^ns^	3.47*	1.3^ns^	3.33*
*F* (Storage time)	35.26***	70.9***	37.54***	124.92***	8.49***
*F* (Salicylic acid × Storage time)	77.5***	89.15***	58.7***	385.09***	72.72***

*Note*: Different letters in the same column indicate statistical differences at *p* ≤ .05.

Abbreviation: ns, not significant.

* *p* ≤ .05.

** *p* ≤ .01.

*** *p* ≤ .001.

### 3.4 Relationships among quality attributes, organic acids, phenolic compounds, and calcium chloride during storage

**FIGURE 2:**
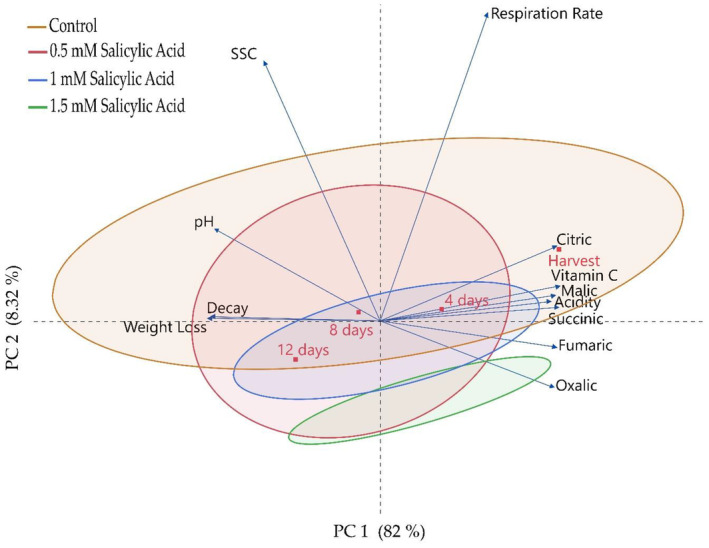
Identification of the effect of postharvest salicylic acid application on quality characteristics and organic acid contents of blackberry fruits by principal component analysis (PCA). SSC, soluble solid contents.

In this study, the effects of salicylic acid application on the quality and some biochemical properties of blackberry fruits during storage were revealed using principal component (PC), heatmap, and correlation statistical analyses. Principal component analysis (PCA) is a statistical evaluation that explains the research results from different scientific perspectives. According to the PCA analysis, the correlation rate indicating the effect of salicylic acid application on organic acids and quality characteristics was determined to be 90.32% (PC1 + PC2) with two principal components (Figure 2). When the correlation between applications was examined, it was found that the 1.5 mM dose showed a significant difference compared to the other applications. In the PCA plane, it was determined that the changes in the ratios of organic acids and vitamin C generally showed parallelism, whereas there was an inverse relationship with pH. While organic acids were generally at high levels at the time of harvest, a decrease was determined depending on the storage period.

**FIGURE 3:**
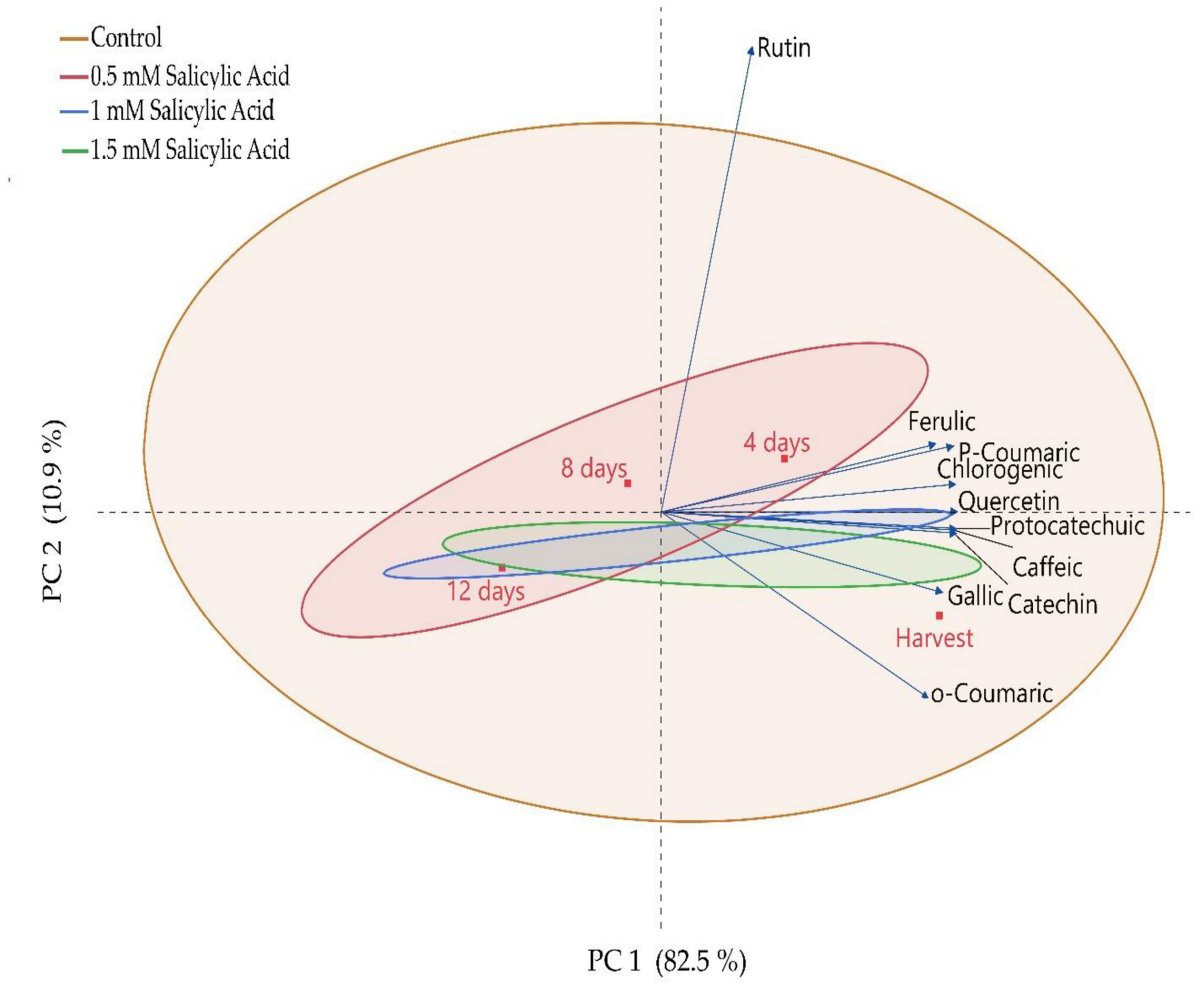
Identification of the effect of postharvest salicylic acid application on phenolic compounds of blackberry fruits by principal component analysis (PCA).

It has been determined that phenolic compounds generally show a decrease during the storage period and that the 1.5 mM dose of salicylic acid application, in particular, largely prevented this decrease. When examining the correlation between the applications and the storage period, it was found that there was a 93.4% (PC1 + PC2) identification with two principal components (Figure 3). Among the phenolic compounds, rutin was found to be dominant and exhibited a different change compared to other phenolics.

**FIGURE 4:**
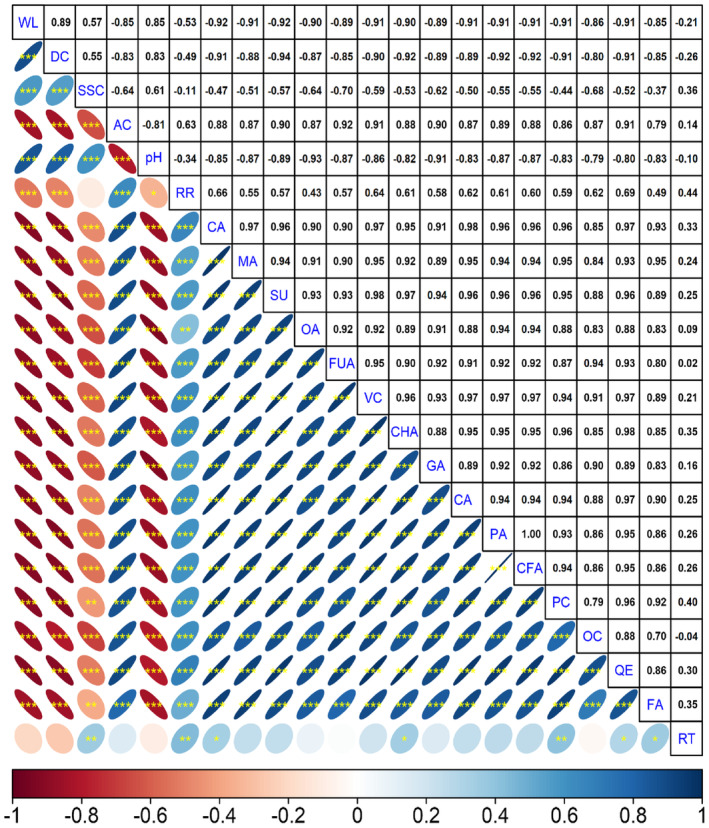
Intercorrelation among physicochemical properties of blackberry fruits. The color gradient ranging from red to blue represents correlation values between −1 and + 1. *, **, and *** denote significance levels at *p* ≤ .05, *p* ≤ .01, and *p* ≤ .001, respectively. AC, acidity; CA, catechin; CA, citric; CFA, caffeic; CHA, chlorogenic; DC, decay; FA, ferulic; FUA, fumaric; GA, gallic; MA, malic; OA, oxalic; OA, *o*‐coumaric; PA, protocatechuic; PA, *p*‐coumaric; QE, quercetin; RR, respiration rate; RT, rutin; SSC, soluble solid contents; SU, succinic acid; VC, vitamin C; WL, weight loss.

According to Pearson correlation analysis, a statistically significant positive correlation (.89) was found between weight loss and decay rate at the *p* ≤ .001 level (Figure 4). The study revealed that there were generally positive correlations among organic acids, and the same was observed among phenolic compounds. Generally, negative correlations were determined between weight loss and decay rate with organic acids. The highest negative correlation was found between citric acid and weight loss (*p* ≤ .001, *r* = .92). The highest negative correlation with the decay rate was formed by succinic acid (*r* = .94).

**FIGURE 5:**
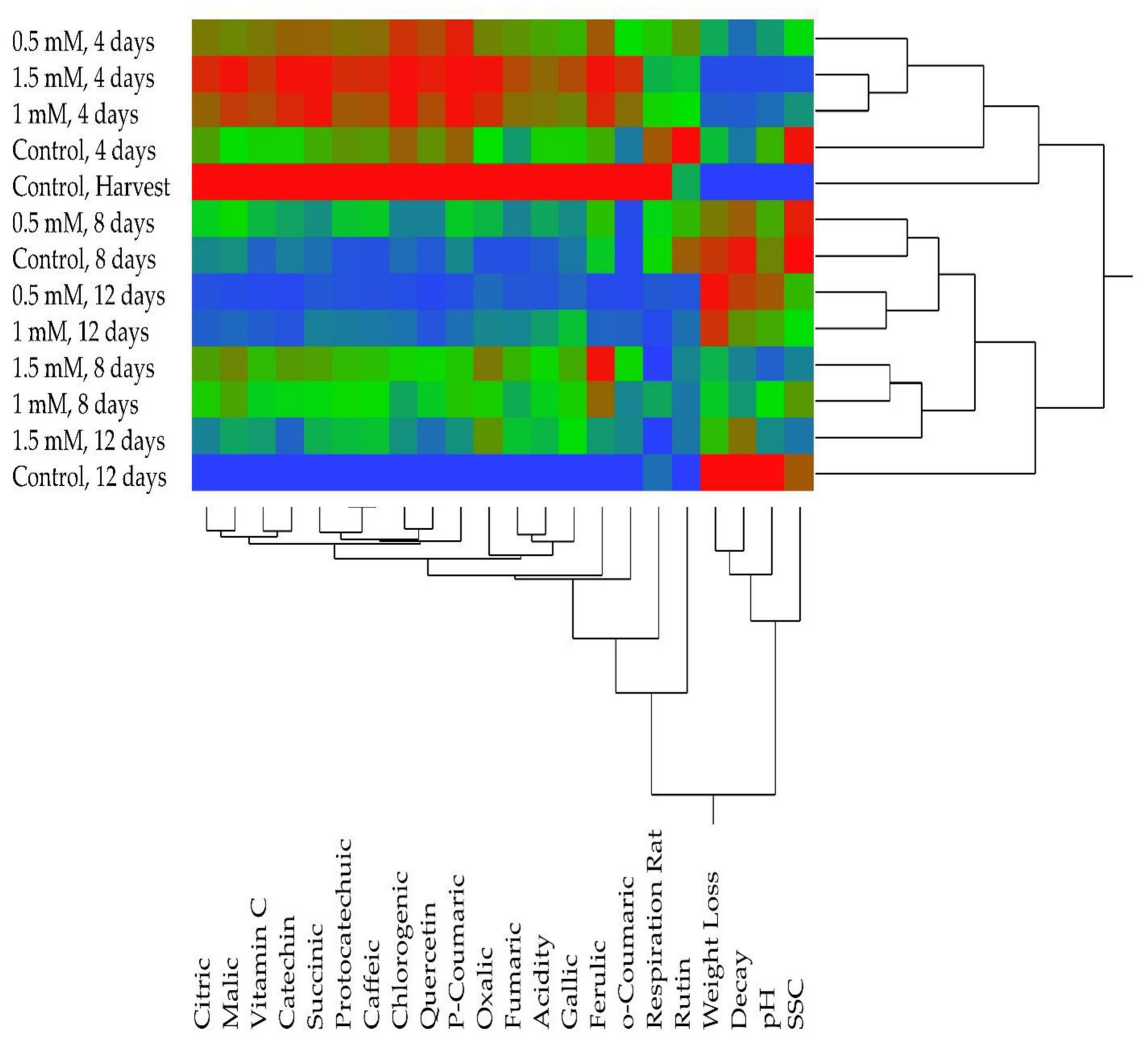
Exploring the relationship among quality properties, organic acid, and phenolic compounds through heatmap analysis.

According to the heatmap analysis, two main groups were formed: the first group included the harvest and the 4th day of storage, while the second group included the 8th and 12th days of storage (Figure 5). When looking at the grouping between fruit quality parameters and organic acids and phenolic compounds, it was observed that quality parameters were in the first group, while phenolic compounds and organic acids were in the second group. Organic acids and phenolic compounds were found at their highest levels at the time of harvest and showed a decreasing trend during the storage period. The changes in the respiration rate, phenolic compounds, and organic acids during storage were similar. A positive correlation between respiration rate and these compounds was clearly determined, and it was evident that the breakdown of these compounds occurred due to respiration.

## 4 CONCLUSION

Salicylic acid application reduced weight loss and decay rate during storage of blackberries. Changes in SSC, TA, pH, and respiration rate during storage were influenced by salicylic acid application, with effects varying depending on the application dose. Overall, salicylic acid effectively maintained levels of organic acids and phenolic compounds, with effectiveness varying by concentration and compound type. The study identified 1.50 mM as the most effective dose for salicylic acid application, demonstrating its potential to preserve postharvest fruit quality and mitigate biochemical changes in blackberries.

## AUTHOR CONTRIBUTIONS

**Mustafa Sakaldaş:** Conceptualization (equal); data curation (equal); formal analysis (equal); investigation (equal); methodology (equal); software (equal); visualization (equal); writing – original draft (equal). **Fatih Şen:** Formal analysis (equal); investigation (equal); methodology (equal); resources (equal); software (equal); validation (equal); visualization (equal). **Muttalip Gundogdu:** Conceptualization (equal); investigation (equal); methodology (equal); software (equal); validation (equal); visualization (equal); writing – review and editing (equal). **Erdal Aglar:** Conceptualization (equal); formal analysis (equal); methodology (equal); software (equal); writing – original draft (equal); writing – review and editing (equal).

## CONFLICT OF INTEREST STATEMENT

The authors declare that there is no conflict of interest regarding the publication of this paper.

## Supporting information


Table S1.

## Data Availability

All data generated or analyzed during this study are included in this published article.
