# Supplementary material for: Alterations in quality characteristics and bioactive compounds of blackberry fruits subjected to postharvest salicylic acid treatment during cold storage
Source: Food Sci Nutr. 2024 Sep 24;12(11):9123–35. doi: 10.1002/fsn3.4490 (PMC11606889; doi:10.1002/fsn3.4490)
Supplement: Supplementary file 1 — Table S1. [file FSN3-12-9123-s001.docx]

**Supplementary material**

**Sup. Table 1.** Impact of salicylic acid dosages on blackberry fruit quality throughout storage periods.

| Salicylic acid application | | Weight Loss (%) | Decay (%) | SSC (%) | pH | Acidity (%) | Respiration Rate  (mg CO_2_ kg^-1^ h^-1^) |
| --- | --- | --- | --- | --- | --- | --- | --- |
| Control | | 5.77±1.87a | 13.72±6.81a | 13.03±1.06a | 3.93±0.30a | 0.91±0.11b | 12.24±4.13a |
| SA 0.5 mM | | 5.07±1.80ab | 10.88±5.07ab | 12.00±1.72b | 3.68±0.22b | 0.98±0.13ab | 10.11±2.26ab |
| SA 1 mM | | 3.81±2.11bc | 7.20 ± 4.15b | 11.39 ± 0.58b | 3.52 ± 0.19b | 1.05 ± 0.08a | 9.21 ± 2.30bc |
| SA 1.5 mM | | 2.89 ± 1.82c | 6.69 ± 5.01b | 10.19 ± 0.51c | 3.28 ± 0.12c | 1.07 ± 0.08a | 7.09 ± 2.12c |
| Storage time |  |  |  |  |  |  |  |
| Harvest | | 0.00 ± 0.00d | 0.00 ± 0.00c | 9.12 ± 0.26c | 3.07 ± 0.02c | 1.37 ± 0.02a | 25.22 ± 1.32a |
| Day 4 | | 2.29 ± 0.32c | 3.60 ± 0.42c | 11.19 ± 0.48b | 3.40 ± 0.05b | 1.12 ± 0.01b | 12.73 ± 0.76b |
| Day 8 | | 4.58 ± 0.34b | 10.42 ± 1.26b | 12.27 ± 0.34a | 3.58 ± 0.05b | 0.98 ± 0.02c | 9.29 ± 0.61c |
| Day 12 | | 6.28 ± 0.40a | 14.85 ± 0.92a | 11.51 ± 0.20ab | 3.83 ± 0.09a | 0.91 ± 0.02d | 6.97 ± 0.27d |
| Storage time × Salicylic acid interaction | |  |  |  |  |  |  |
| Harvest | | 0.00 ± 0.00h | 0.00 ± 0.00h | 9.12 ± 0.26g | 3.07 ± 0.02i | 1.37 ± 0.02a | 25.22 ± 1.32a |
| Day 4 | Control | 3.50 ± 0.32ef | 5.02 ± 0.45ef | 12.98 ± 0.74ab | 3.68 ± 0.03c | 1.05 ± 0.02cd | 17.26 ± 0.99b |
|  | SA 0.5 mM | 3.19 ± 0.53f | 4.34 ± 1.00ef | 11.39 ± 1.41cde | 3.43 ± 0.07e | 1.10 ± 0.02bc | 12.20 ± 0.68c |
|  | SA 1 mM | 1.61 ± 0.24g | 3.42 ± 0.27fg | 10.76 ± 0.19def | 3.31 ± 0.04fg | 1.15 ± 0.01b | 11.53 ± 0.32cd |
|  | SA 1.5 mM | 0.85 ± 0.10gh | 1.63 ± 0.17gh | 9.62 ± 0.05fg | 3.18 ± 0.04hi | 1.17 ± 0.02b | 9.92 ± 0.23de |
| Day 8 | Control | 6.07 ± 0.25bc | 16.15 ± 1.33b | 13.67 ± 0.48a | 3.79 ± 0.03bc | 0.89 ± 0.01e | 11.29 ± 0.62cde |
|  | SA 0.5 mM | 5.26 ± 0.52cd | 13.60 ± 0.42cd | 12.86 ± 0.53ab | 3.71 ± 0.02c | 0.98 ± 0.08d | 10.62 ± 0.53cde |
|  | SA 1 mM | 3.68 ± 0.31ef | 6.40 ± 1.41e | 11.98 ± 0.14bcd | 3.56 ± 0.07d | 1.02 ± 0.02d | 9.66 ± 0.51ef |
|  | SA 1.5 mM | 3.31 ± 0.47ef | 5.54 ± 0.50ef | 10.57 ± 0.14def | 3.27 ± 0.04gh | 1.04 ± 0.01cd | 5.59 ± 0.15i |
| Day 12 | Control | 7.73 ± 0.12a | 20.00 ± 0.51a | 12.45 ± 0.04abc | 4.32 ± 0.03a | 0.80 ± 0.02f | 8.18 ± 0.32fg |
|  | SA 0.5 mM | 6.77 ± 0.52ab | 14.71 ± 0.85bc | 11.76 ± 0.22b-e | 3.90 ± 0.02b | 0.88 ± 0.02ef | 7.50 ± 0.36gh |
|  | SA 1 mM | 6.13 ± 0.71bc | 11.79 ± 1.30d | 11.42 ± 0.04cde | 3.71 ± 0.04c | 0.97 ± 0.00d | 6.43 ± 0.21hi |
|  | SA 1.5 mM | 4.50 ± 0.70de | 12.90 ± 0.94cd | 10.39 ± 0.23efg | 3.40 ± 0.04ef | 0.99 ± 0.02d | 5.76 ± 0.20i |
| ANOVA |  |  |  |  |  |  |  |
| F (Salicylic acid) | | 5.47** | 4.6** | 14.55*** | 18.99*** | 5.26** | 6.84*** |
| F (Storage time) | | 35.33*** | 35.05*** | 5.78** | 12.77*** | 43.73*** | 69.42*** |
| F (Salicylic acid x Storage time) | | 29.68*** | 56.37*** | 7.19*** | 71.89*** | 26.05*** | 81.15*** |

Different letters in the same column indicates statistical differences at *p*≤0.05. **, *** indicates *p*≤0.01 and 0.001, respectively.
